# Correction to “Assessment of the Reaction Location of Skeletal 1-Butene Isomerization over Ferrierite”

**DOI:** 10.1021/acscatal.5c00777

**Published:** 2025-02-10

**Authors:** Pawel A. Chmielniak, Karoline L. Hebisch, Urim Pearl Kim, Jeffrey C. Kenvin, Carsten Sievers

## Statement Regarding Authors

This correction to “Assessment of the Reaction Location of Skeletal 1-Butene Isomerization over Ferrierite” was only prepared by Pawel A. Chmielniak, Karoline L. Hebisch, Urim Pearl Kim, and Carsten Sievers. Jeffrey C. Kenvin was unavailable for comment.

## Correction

The authors acknowledge an error in eq 5 in the Experimental Section of the original paper. The fractional mass uptake of the adsorbate *M*_*t*_ as a function of time *t*, diffusivity *D*, and characteristic length *R*, here reproduced as eq 1, was written as
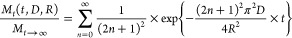
1However, for long times *t*, this equation clearly reaches zero and not unity. The correct form of eq 1 should be presented as follows:
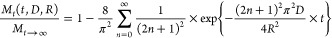
2This error does not change the result, because eq 2 was correctly implemented in the Python subroutines. This can be checked in the Python code which was provided in the Supporting Information of the original publication. The fit for a narrow distribution of characteristic lengths, weighted by a weight factor *w*(*R*) such that ∑_*R*_*w*(*R*) = 1, was obtained from eq 3:
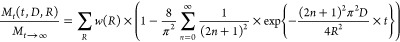
3

